# Restless Legs Syndrome as the Initial Presentation of Multiple Sclerosis

**DOI:** 10.1155/2013/290719

**Published:** 2013-12-19

**Authors:** Ceyla Irkec, Doga Vurallı, Sebnem Karacay Ozkalaycı

**Affiliations:** Department of Neurology, Faculty of Medicine, Gazi University, Beşevler, 06500 Ankara, Turkey

## Abstract

The restless legs syndrome (RLS) is a common central nervous system disorder. It is characterized by complaints of unpleasant sensation in the legs occurring during periods of leg inactivity which worsen or only occur in the evening or at night and relieved partially or totally by movement. The RLS may be idiopathic or due to secondary causes. It is associated with several pathological or physiological conditions. Iron metabolism and dysfunctions of the dopaminergic system are the most important factors in the pathophysiology. There are several studies suggesting multiple sclerosis as one of the causes of symptomatic RLS. Here, we report a case of RLS as the initial presentation of MS. The sudden onset of RLS symptoms in our patient suggested the possibility of an underlying cause. His diagnostic evaluation excluded other causes of RLS and his clinical course suggested that RLS was due to MS. MS with the spinal cord involvement is mostly associated with RLS, but any lesion in the hypothalamic-spinal connection may cause disinhibition of lower spinal levels, resulting in RLS. RLS as the initial presentation of MS reflects that the pathophysiology of RLS in MS is related to inflammatory demyelination rather than axonal degeneration.

## 1. Introduction

The restless legs syndrome (RLS) is a common central nervous system disorder with a prevalence in the general population ranging between 2.5 and 15% [1]. It is characterized by complaints of unpleasant sensation in the legs occurring during periods of leg inactivity which worsen or only occur in the evening or at night and relieved partially or totally by movement [2]. The diagnosis of RLS is established by the clinical features based on the criteria of International Restless Legs Syndrome Study Group (IRLSSG) [3]. The RLS maybe idiopathic or due to secondary causes. It is associated with several pathological or physiological conditions such as iron deficiency, diabetes mellitus, peripheral neuropathies, Parkinson's disease, essential tremor, spinocerebellar ataxias, myelopathies, renal failure, rheumatoid arthritis, and pregnancy [4–6]. Iron metabolism and dysfunctions of the dopaminergic system are the most important factors in the pathophysiology [7]. There are several studies suggesting multiple sclerosis as one of the causes of symptomatic RLS [6, 8–10]. Here, we report a case of RLS as the initial presentation of MS.

## 2. Case Report

A 44-year-old man presented with a sudden onset of lower extremity paresthesias, with an urge to move his legs when he rests in bed or sits for a long time. The patient was questioned regarding the clinical symptoms of RLS based on the IRLSSG criteria. When he rests in bed or sits for a long time, he had unpleasant sensation in the legs and he had an urge to move his legs. His complaints worsened in the evening and especially occur when he lies in bed trying to sleep at night. He had to walk for a while to relieve these complaints. His examination was normal except brisk lower extremity deep tendon reflexes.

**Figure 1:**
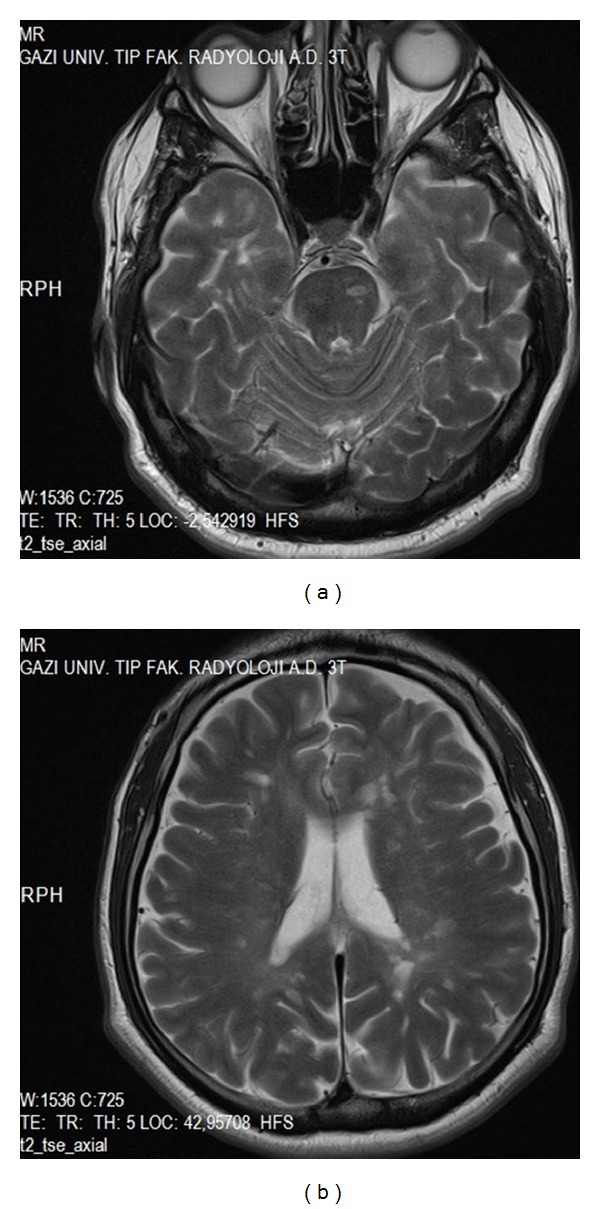
(a) Demyelinating periventricular lesions, (b) demyelinating lesions in the pons, T2 weighted axial section, and brain MRI.

**Figure 2:**
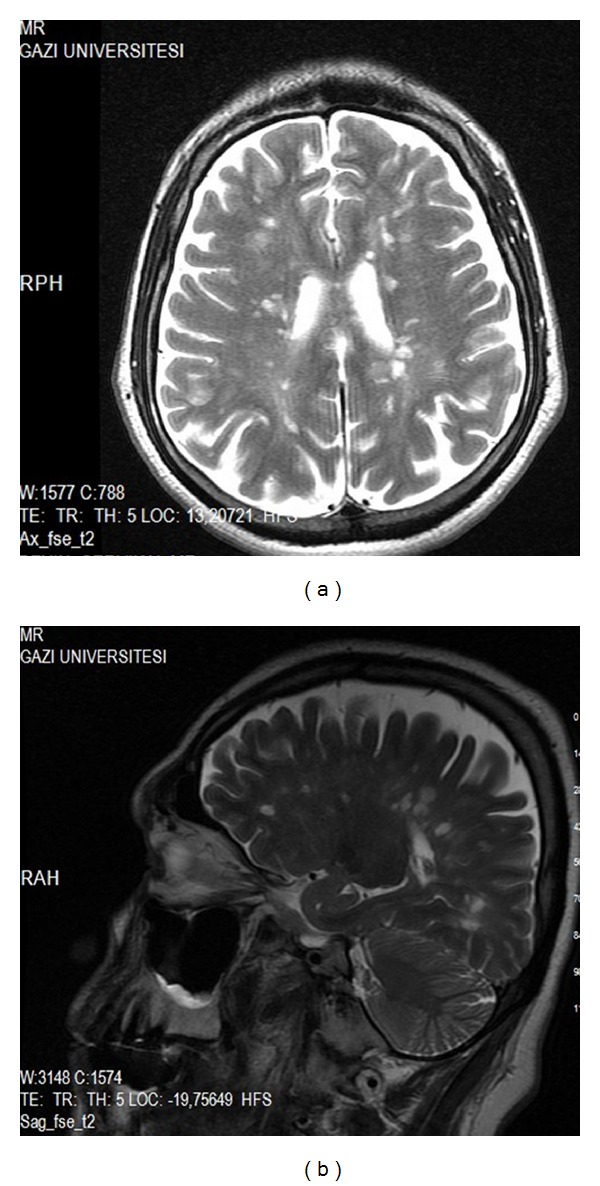
(a) Demyelinating periventricular lesions, T2 weighted axial section, (b) demyelinating periventricular lesions, T2 weighted sagittal section, and control brain MRI.

**Figure 3:**
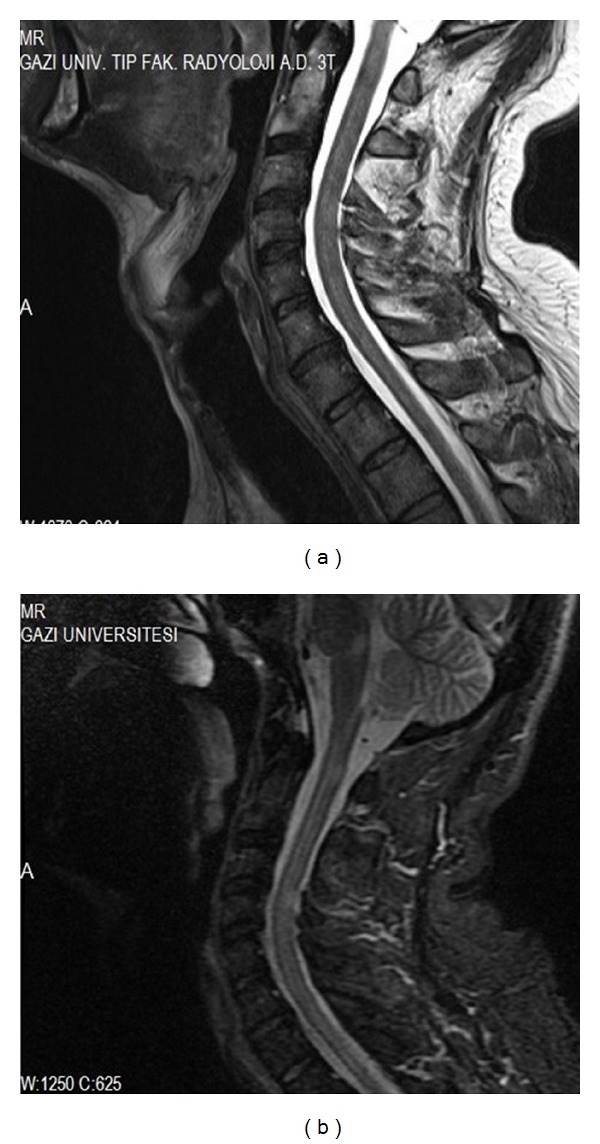
(a) Normal cervical MRI, T2 weighted sagittal section, (b) demyelinating posterior and posterolateral cord lesions in C1, C4, and C5-6 intervertebral disc levels and in upper thoracic segments, T2 weighted sagittal section, and control cervical MRI.

MRI of the brain revealed periventricular and pons plaques. (Figures 1(a) and 1(b)) His cervical (Figure 3(a)) and lumbar MRI was normal. His vitamin B12, vitamin E and D levels, serum iron, iron-binding capacity, and ferritin were all within normal limits and autoantibody tests such as ANA, anti-ds DNA, ANCA, anti-SSA, anti-SSB, and antiphospholipid antibodies were negative. He did not have any drug intake (such as dopamine antagonists, antidepressants, and lithium) associated with RLS. Posterior tibial somatosensory evoked potentials showed prolonged P1 and P2 latencies and central conduction time on the left side. Pramipexole was prescribed and increased to a dose of 0.5 mg/day. Four months after his initial presentation, he developed blurred vision in the right eye. Neurological examination revealed right optic disc edema and diminished visual acuity. Visual evoked potential showed prolonged P100 latency on the right side. 1000 mg methylprednisolone was given for five days and his blurred vision was resolved within 2 weeks. One month later he had right hemiparesis confirming a diagnosis of clinically definite MS. Neurological examination using the manual muscle test revealed a right arm and right motor weakness of 4/5 on the Medical Research Council (MRC) scale, deep tendon reflexes on the right were 3+, and a Babinski response on the right without clonus was present. His control brain MRI demonstrated demyelinating plaques in the supraventricular and periventricular white matter, pons, and both middle cerebellar pedincles (Figures 2(a) and 2(b)) and his control cervical MRI showed demyelinating lesions in C1, C4, and C5-6 intervertebral disc levels and in upper thoracic segments especially placed in posterior and posterolateral cord (Figure 3(b)). Interferon beta 1a treatment was started.

## 3. Discussion

Restless legs syndrome is mostly idiopathic, but it may also be due to secondary causes, in our case multiple sclerosis. Deriu et al. found RLS prevalence in MS patients 5 times higher than that in the control group. Several studies reported the prevalence of RLS in MS patients as being higher than 30% [8, 9]. It is previously reported that older age, severe disability, and cervical cord damage are related to higher frequency of RLS in MS patients [6, 8, 11] and RLS is more likely to be seen in the advanced stages of MS [6, 12]. However, in our case, it is seen in the very early stage of the disease. Iron metabolism and dysfunctions of the dopaminergic system are accused in the pathophysiology [7]. Low brain iron levels are even accused in the pathophysiology of idiopathic RLS [7]. Iron is a cofactor in CNS myelination; thus, its deficiency may play a role in demyelination [13]. MS has been also associated with the abnormal accumulation of iron in the basal ganglia and thalamus [14]. However CSF iron concentrations are reported to be increased in chronic progressive MS [15]; since in our case RLS is the initial presentation of MS, most probably RLS is caused by the demyelination process in MS as emphasized before [16], not due to axonal degeneration related to iron accumulation. Another possible reason of RLS is dopaminergic neurotransmitter dysfunction which is thought to be caused by hyperexcitability of the spinal locomotor generator due to impaired descending cerebro-spinal inhibitory pathway [17]. MS with the spinal cord involvement is mostly associated with RLS but any lesion in the hypothalamic-spinal connection (A11 hypothalamic area to the dorsal and intermediolateral spinal nuclei) may cause disinhibition of lower spinal levels, resulting in RLS [18].

In the recent years, several studies have reported an increased incidence of RLS in patients with MS [8, 19]. The patient described above fulfilled all of the diagnostic criteria of RLS. The sudden onset of RLS symptoms suggested the possibility of an underlying cause. His diagnostic evaluation excluded other causes of RLS and his clinical course suggested that RLS was due to MS RLS as the initial presentation of MS, reflects that the pathophysiology of RLS in MS is related to inflammatory demyelination rather than axonal degeneration.
